# Anakinra Activates Superoxide Dismutase 2 to Mitigate Inflammasome Activity

**DOI:** 10.3390/ijms22126531

**Published:** 2021-06-18

**Authors:** Marilena Pariano, Stefania Pieroni, Antonella De Luca, Rossana G. Iannitti, Monica Borghi, Matteo Puccetti, Stefano Giovagnoli, Giorgia Renga, Fiorella D’Onofrio, Marina M. Bellet, Claudia Stincardini, Maria Agnese Della-Fazia, Giuseppe Servillo, Frank L. van de Veerdonk, Claudio Costantini, Luigina Romani

**Affiliations:** 1 Department of Medicine and Surgery, University of Perugia, 06132 Perugia, Italy; marilena.pariano@gmail.com (M.P.); pistefy@yahoo.it (S.P.); antonelladeluca80@hotmail.it (A.D.L.); r.iannitti@srfarmaceutici.com (R.G.I.); monicaborghi@live.com (M.B.); rengagiorgia@gmail.com (G.R.); donofrio.fiorella@libero.it (F.D.); marinamaria.bellet@unipg.it (M.M.B.); claudiastincardini@gmail.com (C.S.); mariaagnese.dellafazia@unipg.it (M.A.D.-F.); giuseppe.servillo@unipg.it (G.S.); costacla76@gmail.com (C.C.); 2 Department of Pharmaceutical Science, University of Perugia, 06132 Perugia, Italy; matteo.puccetti@gmail.com (M.P.); stefano.giovagnoli@unipg.it (S.G.); 3 Department of Medicine, Radboud University Medical Center, 6525 GA Nijmegen, The Netherlands; frank.vandeVeerdonk@radboudumc.nl

**Keywords:** NLRP3 inflammasome, anakinra, superoxide dismutase 2, oxidative stress, cystic fibrosis, chronic granulomatous disease

## Abstract

Inflammasomes are powerful cytosolic sensors of environmental stressors and are critical for triggering interleukin-1 (IL-1)-mediated inflammatory responses. However, dysregulation of inflammasome activation may lead to pathological conditions, and the identification of negative regulators for therapeutic purposes is increasingly being recognized. Anakinra, the recombinant form of the IL-1 receptor antagonist, proved effective by preventing the binding of IL-1 to its receptor, IL-1R1, thus restoring autophagy and dampening NLR family pyrin domain containing 3 (NLRP3) activity. As the generation of mitochondrial reactive oxidative species (ROS) is a critical upstream event in the activation of NLRP3, we investigated whether anakinra would regulate mitochondrial ROS production. By profiling the activation of transcription factors induced in murine alveolar macrophages, we found a mitochondrial antioxidative pathway induced by anakinra involving the manganese-dependent superoxide dismutase (MnSOD) or SOD2. Molecularly, anakinra promotes the binding of SOD2 with the deubiquitinase Ubiquitin Specific Peptidase 36 (USP36) and Constitutive photomorphogenesis 9 (COP9) signalosome, thus increasing SOD2 protein longevity. Functionally, anakinra and SOD2 protects mice from pulmonary oxidative inflammation and infection. On a preclinical level, anakinra upregulates SOD2 in murine models of chronic granulomatous disease (CGD) and cystic fibrosis (CF). These data suggest that protection from mitochondrial oxidative stress may represent an additional mechanism underlying the clinical benefit of anakinra and identifies SOD2 as a potential therapeutic target.

## 1. Introduction

The inflammasomes are cytosolic multimeric protein complexes that assemble upon stimulation by a variety of microbial or environmental cues to generate active interleukin-1 (IL-1) family proteins and drive an inflammatory response [1]. The importance of inflammasome in sensing molecular patterns and perturbations in cytoplasmic homeostasis, or homeostasis-altering molecular processes [2], is increasingly being recognized. However, a dysfunctional inflammasome activation may lead to pathogenic conditions [3], thus making the control of inflammasome assembly and activation a critical checkpoint for physiological homeostasis. It is recognized that the activation of the inflammasome requires two distinct steps [4]. A first priming step mediated by pathogen-associated molecular patterns or cytokines upregulates the levels of inflammasome components, that are safely present at low levels under normal conditions. Then, a second activation step mediated by a wide range of stimuli ignites the oligomerization of the inflammasome complex via as-yet unclear molecular events, finally resulting in the production of IL-1β [4]. It follows that interfering with either one of the two steps, that is the priming of the inflammasome and oligomerization of the complex, may provide a valid therapeutic option for pathological conditions characterized by hyperactivation of the inflammasome.

The IL-1 receptor antagonist (IL-1Ra) is a member of the IL-1 family that binds to IL-1 receptor 1 (IL1R1) to prevent its activation by IL-1 [5]. Anakinra, the recombinant version of IL-1Ra, from which it differs for the presence of an N-terminal methionine and the absence of glycosylation, has proved effective for a variety of IL-1-driven inflammatory pathologies [5] that expand the currently approved indications for the treatment of rheumatoid arthritis and cryopyrin-associated periodic syndrome [6,7]. In agreement with these findings, we have recently demonstrated that anakinra can reduce inflammation in chronic granulomatous disease (CGD), a congenital immunodeficiency characterized by mutations in components of the NADPH oxidase with reduced generation of reactive oxidative species (ROS), resulting in defective microbial killing by phagocytes and increased susceptibility to infection [8]. We have similarly shown that anakinra can ameliorate inflammation in cystic fibrosis (CF), a genetic disease characterized by mutations in the chloride/bicarbonate channel named cystic fibrosis transmembrane conductance regulator, resulting in dehydrated surface fluids and an increased susceptibility to microbial colonization and infection [9]. In both diseases, anakinra was able to reduce the inflammatory pathology, not only by preventing the effects of IL-1, but also by decreasing its synthesis via inhibition of the NLRP3 inflammasome. One likely mechanism for the inhibitory activity of anakinra was the prevention of inflammasome priming induced upon IL-1 binding to IL-1R. In addition, in both diseases, the downregulation of the levels and activity of the NLRP3 inflammasome by anakinra was strictly intertwined with its ability to restore defective autophagy [8,9]. As a physiological process that removes damaged or dangerous components to re-establish homeostasis, autophagy is emerging as a critical process for the regulation of inflammasome activity [10]. In particular, a non-selective form of autophagy implicated in the degradation of damaged mitochondria, or mitophagy, was shown to negatively regulate NLRP3 inflammasome by reducing the levels of mitochondrial ROS (mtROS) that accumulates in conditions of stress [4,10]. Although the generation of mtROS is considered a critical upstream event in the activation of the inflammasome, whether anakinra can regulate mtROS production has remained unexplored.

Based on these premises, this study has assessed whether and how anakinra could interfere with the activation of NLRP3 inflammasome by mtROS. We found that anakinra promoted mitochondrial biogenesis and ROS-detoxification, and identified manganese-dependent superoxide dismutase (MnSOD) or SOD2 as a central molecule involved in mediating these effects. These results further extend the molecular mechanisms underlying the beneficial effects of anakinra in ameliorating the inflammatory response in pathological conditions characterized by dysfunctional inflammasome activation and abnormal IL-1 production.

## 2. Results

### 2.1. Anakinra Prevents Mitochondrial Oxidative Stress via PGC1alpha and SOD2

**Figure 1:**
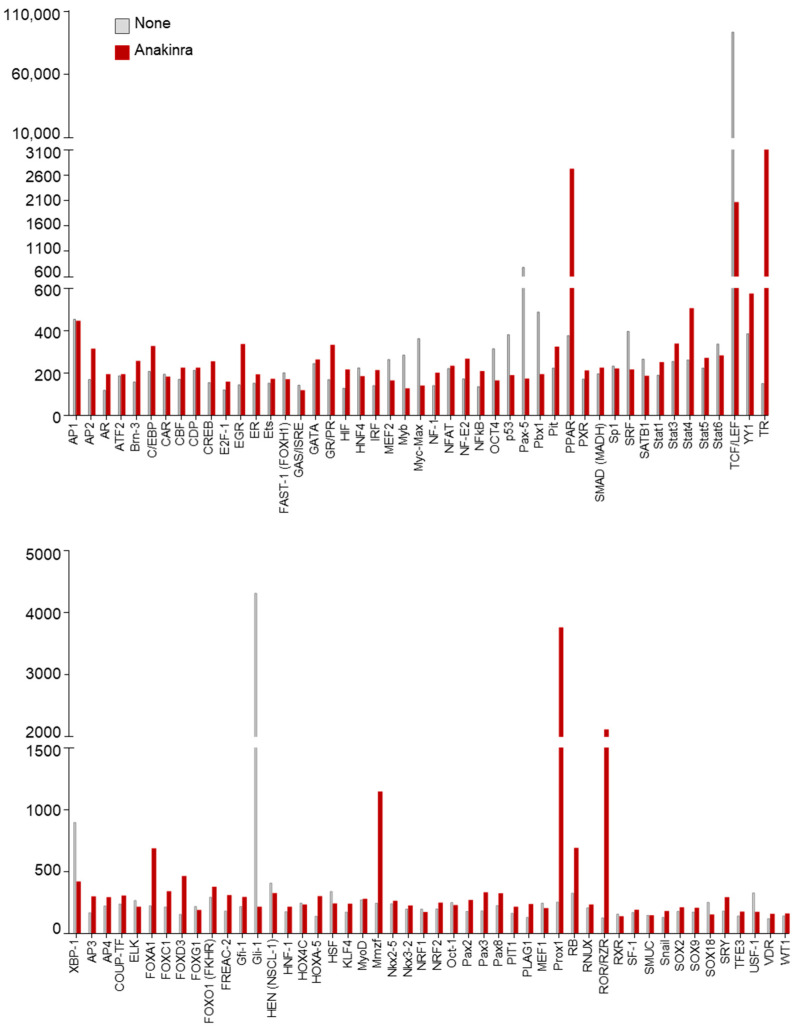
Transcription factors activation profiling analysis of alveolar macrophages isolated from C57BL/6 mice and treated or not with 10 µg/mL anakinra for 1 h.

In a preliminary analysis to unravel the mechanistic events underlying the protective effects of anakinra against inflammasome activation, we profiled the transcription factors activated in alveolar macrophages purified from the lungs of C57BL/6 mice treated or not with anakinra for 1 h (Figure 1).

**Figure 2:**
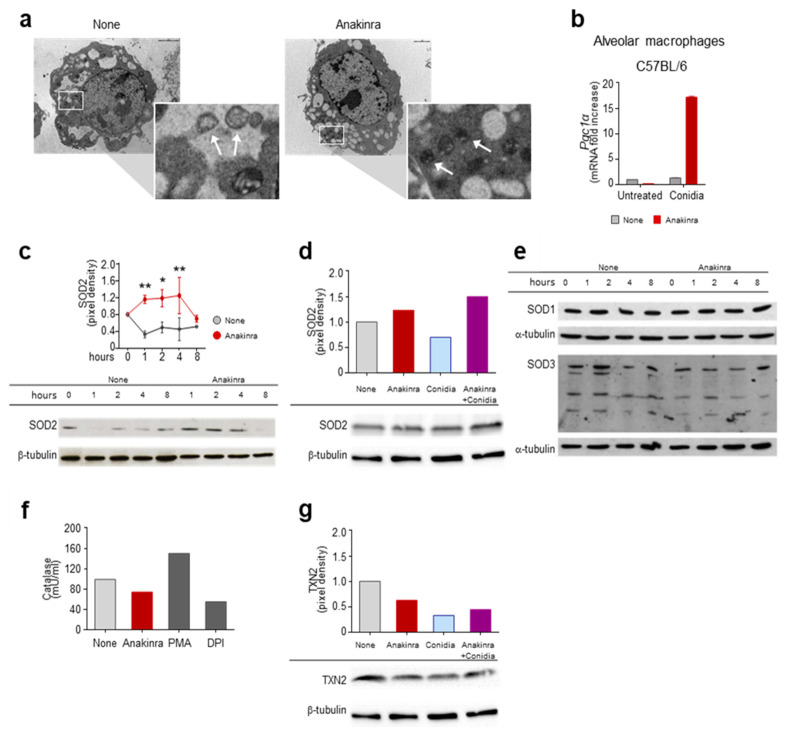
Anakinra spares mitochondria from oxidative damage by inducing PGC1α and SOD2: (**a**) Scanning electron microscopy of RAW 264.7 cells exposed to anakinra for 4 h. Images were taken with the Philips XL30 field emission scanning electron microscope. Note the presence of autophagic vacuoles engulfing enlarged mitochondria (arrows in the inset) in untreated cells as opposed to intact mitochondria (arrows in the inset) in anakinra-treated cells (indicated by squares and magnified in the insets). (**b**) Expression of the peroxisome proliferator-activated receptor coactivator 1α (*Pgc1α*) by RT-PCR in alveolar macrophages exposed to anakinra in the presence or not of *Aspergillus* conidia. (**c**–**g**) RAW 264.7 cells were treated with anakinra (**c**–**g**) and/or *Aspergillus* conidia (**d**,**g**) for 4 h (**d**,**f**,**g**) or the indicated times (**c**,**e**) for the expression of SOD2 (**c**,**d**), SOD1 and SOD3 (**e**), catalase activity (**f**) and the expression of TXN2 (**g**). PMA: Phorbol 12-myristate 13-acetate; DPI: Diphenyleneiodonium. * *p* < 0.05, ** *p* < 0.01, two-way ANOVA, none vs. anakinra.

As a result, we found that, among others, peroxisome proliferation-activated receptor (PPAR), a family of ligand-activated transcription factors involved in energy homeostasis [11], including regulation of mitochondrial function, was activated by anakinra (Figure 1). To more directly test whether anakinra could affect mitochondrial dynamics, we treated RAW264.7 cells with anakinra for 4 h and analyzed mitochondrial morphology in a field emission scanning electron microscope. We found that, while untreated cells showed autophagic vacuoles engulfing enlarged mitochondria, the treatment with anakinra preserved intact mitochondria, suggesting that anakinra could spare mitochondria from oxidative damage (Figure 2a).

To gain an insight into the mechanism of action of anakinra, we treated alveolar macrophages with *Aspergillus* conidia [8,9], and evaluated the expression of PPAR Gamma Coactivator-1alpha (PGC-1α), a master regulator of mitochondrial biogenesis and function [12]. As shown in Figure 2b, the treatment with anakinra increased the levels of PGC1α in response to conidia. It is of note that PGC1α is known to protect from oxidative stress and mitochondrial dysfunction by increasing the levels of antioxidant enzymes, such as SOD2, catalase, thioredoxin-2 (TXN-2), and other effector molecules [12]. We thus evaluated the expression of antioxidant enzymes and found that anakinra increased the levels of SOD2 protein expression in RAW 264.7 cells in a time-dependent manner (Figure 2c), as well as affecting exposure to *A. fumigatus* conidia (Figure 2d). Other antioxidants, such as SOD1, SOD3 (Figure 2e), catalase (Figure 2f) and TXN-2 were not induced by anakinra (Figure 2g).

These data indicate that anakinra protects mitochondria from oxidative stress, likely by engaging the PGC1α pathway and inducing SOD2.

### 2.2. Anakinra Prevents SOD2 Degradation via USP36-COPS3

**Figure 3:**
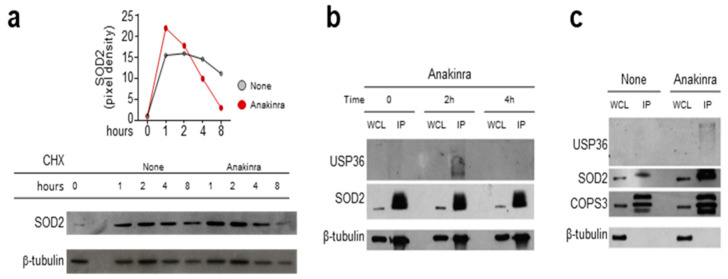
Anakinra promotes the interaction between SOD2, USP36, and COP9 signalosome: (**a**) Western blot analyses of RAW 264.7 cells exposed or not (none) to 10 μg/mL anakinra in the presence of cyclohexamide (CHX) and evaluated at the indicated times for SOD2 protein level. Relative densitometric evaluation was reported. (**b**) RAW 264.7 cell lysates obtained at 0, 2 and 4 h following anakinra exposure were immunoprecipitated using anti-SOD2 antibody and subsequently revealed for binding to the specific deubiquitinating protein USP36. SOD2 was used as IP control. (**c**) Cell lysates from RAW 264.7 cells treated with anakinra were immunoprecipitated using COPS3 antibody and were revealed for binding to both USP36 and SOD2. COPS3 was used as IP control. WCL: whole cell lysate; IP: immunoprecipitation.

Based on these results, we further analyzed the molecular events underlying the regulation of SOD2 by anakinra. Culturing cells in the presence of anakinra and the protein synthesis inhibitor cyclohexamide resulted in higher levels of SOD2 at early time points, followed by degradation at later time points (Figure 3a), suggesting that anakinra promotes an early sustain of SOD2 levels. It is known that USP36 is able to reduce the ubiquitination level of SOD2, thus increasing the SOD2 half-life [13]. To prove this interaction, we treated RAW264.7 cells with anakinra for 2 and 4 h before immunoprecipitation with an antibody against SOD2. As shown in Figure 3b, USP36 associated with immunoprecipitated SOD2 at 2 h, but not at 4 h, indicating a transient interaction between the two molecules, in line with the transient increase in SOD2 levels. We further characterized the association between SOD2 and USP36, and found that it occurred at the level of the third component of the COP9 signalosome (Figure 3c), as revealed by co-immunoprecipitation with an antibody against COPS3.

Overall, these results suggested that, by mimicking the major intracellular isoform of IL-1Ra [14], anakinra exhibited the ability to bind the third component of the COP9 to deubiquinate SOD2 and sustain its activity.

### 2.3. Anakinra and SOD2 Ameliorates the Oxidative Inflammatory Pathology In Vivo

**Figure 4:**
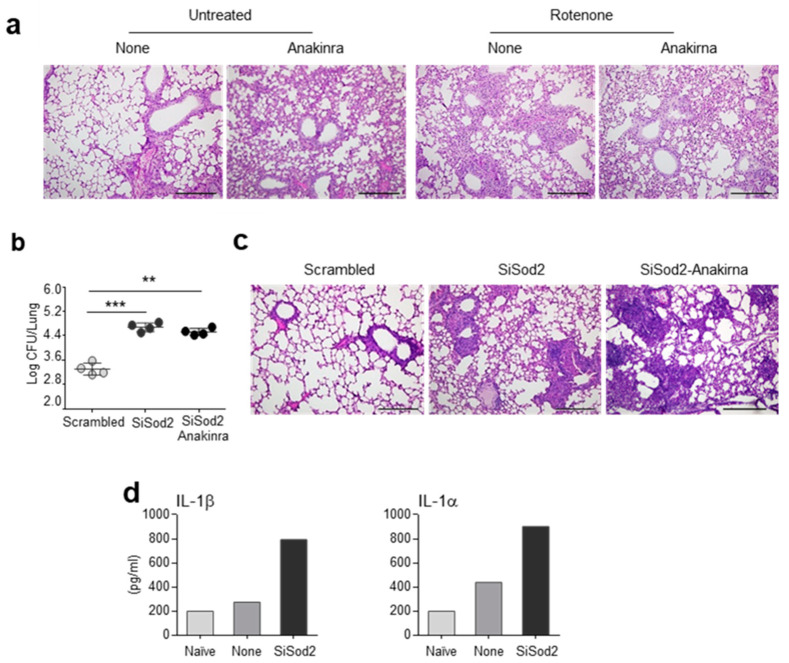
Anakinra and SOD2 ameliorates the inflammatory pathology in vivo: (**a**) C57BL/6 mice were treated with rotenone in the presence or absence of anakinra and evaluated for lung histopathology. (**b**–**d**) C57BL/6 mice were infected intranasally with live *A. fumigatus* conidia and treated with Si*Sod2* or equivalent doses of nonspecific, scrambled SiRNA in a volume of 20 µl of duplex buffer before evaluation of fungal growth (log10 cfu mean ± SEM) (**b**), lung histopathology (**c**) and cytokine quantification (**d**) at 3 dpi. Scale bar, 500 μm. **, *p* < 0.01; ***, *p* < 0.001, one-way ANOVA.

To evaluate the antioxidant role of anakinra and SOD2 in vivo, we first infected C57BL/6 mice intranasally with live *A. fumigatus* conidia in the presence of rotenone, an inhibitor of the electron transport chain in mitochondria that results in the production of mtROS [15]. As shown in Figure 4a, rotenone greatly increased the inflammatory pathology in the lungs of the infected mice, an effect that was mitigated by the concurrent treatment with anakinra. We also evaluated whether SOD2 would protect in vivo from oxidative immunopathology. To this purpose, the C57BL/6 mice were treated intranasally with siRNA against SOD2 before challenging them with *A. fumigatus* conidia. We found that, by decreasing SOD2 activity, both the fungal burden (Figure 4b) and lung inflammation (Figure 4c) worsened, as compared to the mice treated with the scrambled siRNA. Interestingly, the reduced levels of SOD2 resulted in a higher production of IL-1β and IL-1α (Figure 4d), indicating that SOD2, by scavenging mtROS, restrained the activation of the inflammasome. In addition, the siRNA against SOD2 abolished the beneficial effects of anakinra (Figure 4b,c), indicating that the anakinra required a functionally active SOD2 to protect from lung inflammatory pathology induced by *Aspergillus* infection.

These results suggest that anakinra engages SOD2 to reduce both mitochondrial oxidative damage and the production of mtROS, thus dampening the activation of the inflammasome and ameliorating the inflammatory pathology.

### 2.4. Anakinra Activates SOD2 in Murine CGD and CF

**Figure 5:**
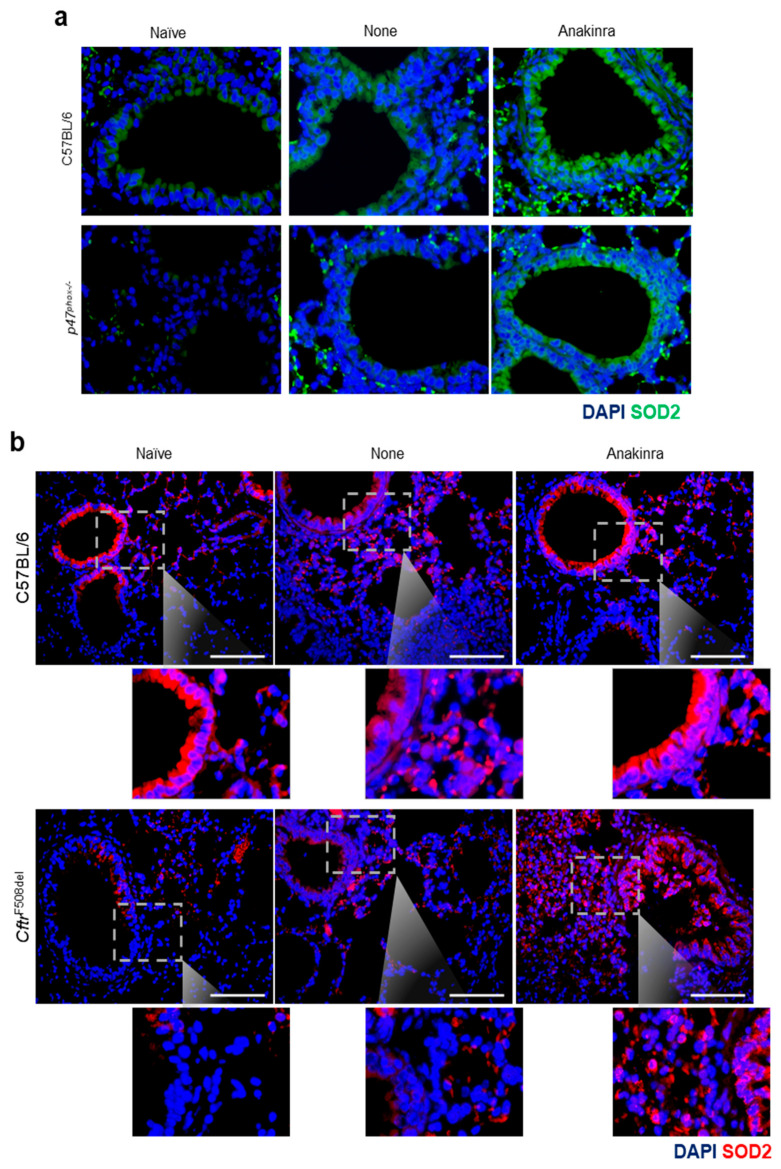
Anakinra increased the lung expression of SOD2 in vivo: C57BL/6 (**a**,**b**), *p47^phox−/−^* (**a**) and *Cftr^F508del^* (**b**) mice were infected with live *Aspergillus* conidia and treated with 10 mg/kg anakinra intraperitoneally for 6 consecutive days before analysis of SOD2 protein expression by immunofluorescence. Scale bar, 100 μm. Naïve: untreated mice, None: mice infected with *Aspergillus* conidia; anakinra: mice infected with *Aspergillus* conidia and treated with anakinra.

Both CGD and CF diseases were characterized by a redox imbalance involving an elevated production of mtROS and mitochondrial dysfunction [16,17,18]. By resorting to a murine model of CGD (*p47^phox−/−^* mice) and CF (*Cftr^F508del^* mice), we explored whether the expression of SOD2 was increased in vivo in the lungs of *Aspergillus*-infected mice and the effect of anakinra treatment on it. We found that SOD2 protein expression was reduced in CGD (Figure 5a) and CF (Figure 5b) mice as compared to control mice, but was promptly restored by treatment with anakinra (Figure 5a,b).

Considering that mtROS scavenging via SOD2 attenuates lung pathology [19], and that SOD2 polymorphisms are associated with bronchial hyperresponsiveness [20], our findings indicate that, by elevating the endogenous levels of SOD2 with anakinra, such a process could be a therapeutic strategy in oxidative stress-induced lung pathology.

## 3. Discussion

The results presented in this study extend our current knowledge of the mechanisms engaged by anakinra to counteract the IL-1-driven inflammatory response. Indeed, anakinra not only prevents the binding of IL-1 to its receptor IL1R1, but can also regulate the levels and activity of the inflammasome and synthesis of IL-1. We have previously shown that anakinra regulated the inflammasome by restoring autophagy in CGD [8] and CF [9]. Herein, we have demonstrated that, by mimicking the effects of intracellular IL-1Ra1 [14], anakinra promoted an antioxidant response via increased expression of mitochondrial SOD2. Mechanistically, anakinra appears to have prevented SOD2 degradation by interfering with the USP36-COPS3 complex rather than inducing its expression. It is becoming increasingly clear that SOD2 is a hub for redox signaling in mitochondria and that its processing of ROS generated in mitochondria broadly affects cellular signaling networks [21]. By preventing mitochondrial damage and the accumulation of ROS, SOD2 has emerged as a critical regulator of inflammasome activation. Indeed, it has already been demonstrated that SOD2 knockdown exacerbated oxidative damage through the assembly and activation of the NLRP3 inflammasome [22]. Moreover, it has been shown that prolonged fasting blunts inflammasome activation by engaging the SIRT3-SOD2 pathway [23]. Activated by inflammatory, metabolic, and stress signals, SOD2 provides protection to an acute insult, but may also contribute to the progressive deterioration of mitochondrial bioenergetics when persistently expressed [24]. Hence, SOD2 activity is tightly regulated. Studies have demonstrated that SOD2 is post-translationally regulated [13,25]. We found that USP36 increased the protein stability of SOD2 in response to anakinra, suggesting that the level of ubiquitination also modulates SOD2 stability. Irrespective of the mechanism by which USP36 is activated in response to anakinra, the overexpression of SOD2 could be of relevance as a decrease in mitochondrial ROS by overexpression of SOD2 protects against mitochondrial oxidative damage and mitochondrial dysfunction [26,27]. In this regard, we have observed that anakinra spared mitochondria of RAW 264.7 cells from oxidative damage, and promoted the expression of the transcriptional co-activator PGC-1α, known to promote mitochondrial biogenesis and ROS-detoxification [28]. In vivo, blocking SOD2 decreased the anti-inflammatory potential of anakinra in the lungs of mice infected with *A. fumigatus*. Thus, like SOD mimetics [29], anakinra may act as a mitochondria-targeted antioxidant agent that can protect against oxidant-related lung disorders and perhaps cardiovascular diseases, in which mitochondrial dysfunction is a major contributor to cardiovascular senescence [30]. Of note, anakinra appears to work as SOD2 mimetic by inducing SOD2 expression rather than complementing its activity by working as an antioxidant itself [31]. The induction of SOD2 was selective, as other antioxidant enzymes did not appear to be modulated by anakinra, as occurs for other SOD2 mimetics, an effect that may be related to the ability of anakinra to interfere with the USP36-COPS3 complex, rather than engaging the Nrf2 pathway [32]. Whatever the case, considering that mitochondrial damage, oxidative stress, and reduced proteostatic capacity are implicated in the progression of aging, along with the pathogenesis of age-related neurodegenerative diseases [33], this may broaden the therapeutic potential of anakinra in human diseases.

In conclusion, our study revealed a novel mechanism employed by anakinra to inhibit IL-1-driven inflammation, and provides additional insights on the therapeutic efficacy of anakinra in pathological conditions characterized by mitochondrial dysfunction and inflammasome activation.

## 4. Materials and Methods

### 4.1. Cells

RAW 264.7 cells were from *ATCC* (ATCC^®^ TIB-71™). Alveolar macrophages from the lungs of C57BL/6 uninfected mice were obtained after 2 h of plastic adherence at 37 °C. Cells were maintained at 37 °C in a humidified incubator in an atmosphere containing 5% CO_2_. Anakinra (Kineret) was diluted in phosphate-buffered saline (PBS) (vehicle).

### 4.2. Transcription Factors Activation Profiling Analysis

Macrophages cells were purified from the lungs of C57BL6 naïve mice and treated with 10ug/mL of anakinra for 1h. Untreated cells were used as negative controls. Nuclear extracts were prepared with the nuclear extract kit (Signosis, Inc., Santa Clara, CA, USA) according to the manufacturer’s instructions. Briefly, the cells were washed twice in PBS and lysed on ice for 10 min in the extraction buffer I with gentle shaking, before being collected from the plates and centrifuged at 15,000 rpm for 3 min at 4 °C. The supernatant (cytoplasmic fraction) was discarded and the pellet resuspended in 250 μL of extraction buffer II and incubated on ice for 2 h with gentle shaking. After the mixture was centrifuged at 15,000 rpm for 5 min at 4 °C, the supernatant containing nuclear protein was collected and ready for assays. Protein concentrations were determined by the Bradford assay (Bio-Rad). Each array assay was performed following the procedure described in the TF activation profiling plate array kit user manual (Signosis, Inc., Santa Clara, CA, USA). First, 10 µg of nuclear extract was incubated with the biotin labeled probe mix at room temperature for 30 min. The activated TFs were bound to the corresponding DNA binding probes. After the protein/DNA complexes were isolated from the unbound probes, the bound probes were eluted and hybridized with the plate pre-coated with the capture oligos. The captured biotin-labeled probes were then detected with Streptavidin–HRP and subsequently measured with the chemiluminescent plate reader.

### 4.3. Scanning Electron Microscopy

RAW 264.7 cells were exposed to 10 μg/mL of anakinra for 4 h and fixed in 2.5% glutaraldehyde in a 0.1 M sodium cacodylate buffer solution (pH 7.4; 20 °C) for 24 h. Thereafter, the specimens were dehydrated in a graded series of ethanol and then critical point dried. After coating with a 30 nm gold layer in a metal sputter coater, the specimens were studied with the Philips XL30 field emission scanning electron microscope.

### 4.4. siRNA Design and Delivery

Predesigned SiRNA against SOD2 (MMC.RNAI.N013671.12.1) was purchased from Integrated DNA Technologies (IDT) (TEMA Ricerca, Italy). Cells were incubated for 24 h (as indicated by preliminary experiments performed at 12, 24, or 48 h) at 37 °C in 5% CO_2_ with specific SiRNA using the Lipofectamine^®^ LTX Reagent, following the manufactures instructions. The effectiveness of silencing specific targets was verified through quantitative RT-PCR analysis at 24 h. For in vivo studies, each mouse was lightly anesthetized by inhaled diethyl ether, then given an intranasal administration of unmodified siRNA (10 µg/kg) or equivalent doses of nonspecific control SiRNA duplex in a volume of 20 μL of duplex buffer (IDT, TEMA ricerca). Intranasal SiRNA was given twice, two days before infection and 3 days after infection. Nonspecific SiRNA duplex were used as controls.

### 4.5. Western Blot Analysis and Immunoprecipitation

Blots of cell lysates were incubated with antibodies against the following proteins: COPS3, Abcam, Cat. No. Ab79698; SOD1 (Novus Biologicals); SOD2, Millipore, Cat. No. 06-984; SOD2, Thermo Fisher Scientific, Cat. No. A21990, Clone 9E2BD2; SOD3: Abcam, Cat. No. Ab83108; TXN2 (Novus Biologicals); USP36, Proteintech, Cat. No. 14783-1-AP. Normalization was performed with α-tubulin or β-tubulin antibodies (α-tubulin, Sigma–Aldrich, Cat. No. T8203, Clone AA13; β-tubulin, Sigma–Aldrich, Cat. No. T4026, Clone TUB2.1) and GAPDH (Cat. No. G8795, Clone GAPDH-71.1). For immunoprecipitation, cells were lysed in an immunoprecipitation buffer containing 150 mM Sodium Chloride (NaCl), 50 mM Tris (pH 8.0), 1% Triton-X100, 0.5% Sodium deoxycholate, 0.1% Sodium dodecylsulphate (SDS), Complete Protease Inhibitor Cocktail (Roche), and PMSF (Roche). SOD2, USP36, and COPS3 were immunoprecipitated by incubation with 1 µg of the specific antibody. The reaction was performed overnight, and either Protein A or Protein G Sepharose 4 Fast Flow (GE Healthcare) were added and incubated for an additional 2 h. Beads were washed and resuspended in Laemmli buffer. Immunoprecipitated proteins were separated by SDS-PAGE and immunoblots were probed with anti-USP36, anti-SOD2, and anti-COPS3 antibodies. Chemiluminescence detection was performed with a LiteAblotPlus chemiluminescence substrate (Euroclone), using the ChemiDoc XRS+ imaging system (Bio-Rad), and quantification was obtained by densitometry image analysis using Image Lab 5.1 software (Bio-Rad).

### 4.6. Catalase Activity

Catalase activity was measured with a colorimetric kit (Thermofisher) according to manufacturer’s instructions.

### 4.7. ELISA and Real-Time PCR

Cytokines were determined by specific ELISA kits (R&D Systems). Real-time RT-PCR was performed using the iCycler iQ detection system (Bio-Rad) and SYBR Green chemistry (Finnzymes Oy, Espoo, Finland). Cells were lysed and the total RNA was extracted using an RNeasy Mini Kit (QIAGEN), and was reverse transcribed with Sensiscript Reverse Transcriptase (QIAGEN) according to the manufacturer’s directions, as described [34]. The mouse primers (5′–3′) were as follows: Pgc1-α: TCTCAGTAAGGGGCTGGTTG and TTCCGATTGGTCGCTACACC. Amplification efficiencies were validated and normalized against Gapdh. The thermal profile for SYBR Green real-time PCR was at 95 °C for 3 min, followed by 40 cycles of denaturation for 30 s at 95 °C, and an annealing/extension step of 30 s at 60 °C. Each data point was examined for integrity through an analysis of the amplification plot. The mRNA-normalized data were expressed as relative cytokine mRNA in treated cells compared to that of unstimulated cells.

### 4.8. Mice, Infections and Treatments

C57BL/6 wild-type (WT) mice aged 8-to-10-weeks old were purchased from Jackson Laboratories (Bar Harbor, Maine). Homozygous *p47^phox−/−^* mice on C57BL/6 background were purchased (Harlan) and bred under specific pathogen-free conditions at the Animal Facility of Perugia University, Perugia, Italy. CF mice homozygous for the F508del-CFTR, which had been backcrossed for 12 generations to the C57BL/6 strain, or in the FVB/129 outbred background (*Cftr^tm1EUR^*, F508del, abbreviated *Cftr^F508del^* mice), were obtained from Bob Scholte, Erasmus Medical Center Rotterdam, The Netherlands [35], and maintained in our animal facility. These mice were provided with a special food consisting of an equal mixture of SRM-A (Arie Blok, Woerden, The Netherlands). Newborn mice were genotyped by cutting a small piece of tail 12 days after birth. Male and female mice were used in all studies. Mice were anesthetized in a plastic cage by inhalation of 3% isoflurane (Forane, Abbott) in oxygen before an intranasal instillation of 2 × 10^7^ *A. fumigatus* (Af293) resting conidia per 20 μL of saline. Quantification of fungal growth was done as described [34]. For histology, paraffin-embedded sections were stained with periodic acid–Schiff (PAS). Mice were treated intraperitoneally (i.p.) with anakinra reconstituted in sterile water (10 mg/kg) and given daily for 6 consecutive days, beginning the day of the infection. In some experiments, mice were treated with 2.6 mg/kg rotenone i.p. from the day of the infection until the end of the experiment. Infections were performed under isoflurane anesthesia, and all efforts were made to minimize suffering. Mouse experiments were performed according to the Italian Approved Animal Welfare Authorization 360/2015-PR and Legislative Decree 26/2014 regarding the animal license obtained by the Italian Ministry of Health lasting for 5 years (2015–2020).

### 4.9. Immunofluorescence

SOD2 immunofluorescence was done on lung sections by staining with anti-Mn-SOD2 (Millipore). DAPI was used to counter stain nuclei. All images were acquired using a BX51 fluorescence microscope (Olympus) with ×40 objective using the analysis image-processing software (Olympus).

### 4.10. Statistical Analysis

GraphPad Prism software 6.01 (GraphPad Software) was used for the analysis. Data are expressed as mean ± SEM. Statistical significance was calculated through a one or two-way ANOVA (Tukey’s or Bonferroni’s post hoc test) for multiple comparisons, and by a two-tailed Student’s t-test for a single comparison. We considered all *p*-values ≤ 0.05 significant. The in vivo groups consisted of 6 mice/groups. The data reported are either representative of at least three experiments (histology, immunofluorescence, and Western blotting), or pooled otherwise.

## Data Availability

The data presented in this study are available on request from the corresponding author.

## Abbreviations

CGD	chronic granulomatous disease
COP9	Constitutive photomorphogenesis 9
COPS3	COP9 Signalosome Subunit 3
CF	Cystic fibrosis
CHX	Cycloheximide
IL-1	Interleukin-1
IL-1Ra	IL-1 receptor antagonist
IL1R1	IL-1 receptor 1
IP	Immunoprecipitation
mtROS	mitochondrial reactive oxidative species
NLRP3	NLR family pyrin domain containing 3
PGC-1α	PPAR Gamma Coactivator-1alpha
PPAR	peroxisome proliferation-activated receptor
ROS	reactive oxidative species
SIRT3	Sirtuin 3
SOD2	superoxide dismutase
TXN-2	Thioredoxin-2
USP36	Ubiquitin Specific Peptidase 36
WCL	Whole Cell Lysate
